# Advances in spatial transcriptomics and related data analysis strategies

**DOI:** 10.1186/s12967-023-04150-2

**Published:** 2023-05-18

**Authors:** Jun Du, Yu-Chen Yang, Zhi-Jie An, Ming-Hui Zhang, Xue-Hang Fu, Zou-Fang Huang, Ye Yuan, Jian Hou

**Affiliations:** 1 Department of Hematology, School of Medicine, Renji Hospital, Shanghai Jiao Tong University, 160 Pujiang Road, Shanghai, 200127 China; 2 School of Medicine, Shanghai Jiao Tong University, Shanghai, 200025 China; 3 Ganzhou Key Laboratory of Hematology, Department of Hematology, The First Affiliated Hospital of Gannan Medical University, Ganzhou, 341000 Jiangxi China; 4 Institute of Image Processing and Pattern Recognition, Shanghai Jiao Tong University, Shanghai, 200240 China; 5 Key Laboratory of System Control and Information Processing, Ministry of Education of China, Shanghai, 200240 China

**Keywords:** Spatial transcriptomics, Tissue heterogeneity, Methodology

## Abstract

Spatial transcriptomics technologies developed in recent years can provide various information including tissue heterogeneity, which is fundamental in biological and medical research, and have been making significant breakthroughs. Single-cell RNA sequencing (scRNA-seq) cannot provide spatial information, while spatial transcriptomics technologies allow gene expression information to be obtained from intact tissue sections in the original physiological context at a spatial resolution. Various biological insights can be generated into tissue architecture and further the elucidation of the interaction between cells and the microenvironment. Thus, we can gain a general understanding of histogenesis processes and disease pathogenesis, etc. Furthermore, in silico methods involving the widely distributed R and Python packages for data analysis play essential roles in deriving indispensable bioinformation and eliminating technological limitations. In this review, we summarize available technologies of spatial transcriptomics, probe into several applications, discuss the computational strategies and raise future perspectives, highlighting the developmental potential.

## Introduction

Human organs and systems are comprised of distinct cell subpopulations whose physiological processes and functions are deeply correlated with their spatial distributions and cellular interactions. To gain a deeper understanding of tissue architecture as well as heterogeneity and to subsequently obtain biological insights into intercellular communication and microenvironment, it is crucial to decipher the disparities among tissue regions and cells in their original spatial context. Previously developed single-cell RNA sequencing (scRNA-seq) [1] has provided comprehensive information about transcriptomes, altering our ability to identify cell subpopulations. However, the segregation of cells while dissociating the tissue destroys cellular spatial information in the original tissue context, which sometimes could be extremely crucial to understanding intricate cellular interaction networks. Moreover, since scRNA-seq was developed in 2009, many limitations have been emerging. For instance, the relatively low efficiency and coverage of RNA transcript capturing may lead to the loss of gene expression information for downstream analysis [2]. Furthermore, certain types of cells may exhibit significant cell variations due to factors such as cell size and cell cycle stage, causing less reliable results. Another challenge of scRNA-seq is the batch effect which also needs to be considered and corrected before subsequent analyses [3]. Additionally, the dissociation protocol of tissue sections may have repercussions on transcriptome and induce transcriptome-wide changes including ectopic expression of genes, causing a contaminating signal and subsequently leading to the misidentification of cell subpopulations [4]. These obstacles are gradually improved with advances in spatial transcriptomics where each cell is assigned a specific and unique spatial label containing spatial coordinates information, allowing for relatively precisely positioning each identified cell subpopulation to the original tissue sections [5]. Employing spatial transcriptomics techniques enables transcriptomic data to be acquired from intact tissue sections and in turn obtains spatial distribution information and elucidates cellular interaction patterns [2].

Although current cutting-edge spatial transcriptomics techniques are confronted with some drawbacks such as relatively low resolution and comparatively insufficient sequencing depth [2], they are extensively utilized in a wide range of biomedical research because of the accelerating capacity to investigate the spatial architecture of normal tissue and tumor. These approaches and platforms have been applied to the adult mouse brain [6], mouse liver [7], human dorsal root ganglia [8] and dorsolateral prefrontal cortex [9], human heart [10], embryonic liver [11], intestine [12] and mammalian testis [13] to reveal tissue architecture and delineate embryonic developmental blueprint and also been employed to lucubrate disease pathogenesis and microenvironment [14–17]. An important part of the disease research is into tumor biology which encompasses pancreatic ductal adenocarcinoma [18], human squamous cell carcinoma [19], breast cancer [20] and cutaneous malignant melanoma [21], etc. These applications provide adequate novel biological insights and clinical relevance to resolving the intrinsic mechanism of tissue dynamics and disease and to remedying or optimizing present medical treatment protocols. Bioinformatics analysis strategies aim at mutual and disparate purposes concerning clustering analysis, data integration, deconvolution, spatially-variable genes identification, etc. For example, early-developed and now commonly-used Seurat [22] can be applied to clustering and gene imputation, and the recently published Tangram [23] tackles deconvolution and also gene imputation.

Spatial transcriptomics technologies have been continuously making significant progress. Multiple technologies have emerged in recent years, and their applications and advantages and disadvantages are comprehensively reviewed. In this article, we summarize the landscapes of available spatial transcriptomics technologies, present the employment of spatial techniques in extensive fields of biomedical research and focus on the status quo of computational strategies of data analysis.

## Development of spatial transcriptomics technologies

**Fig. 1:**
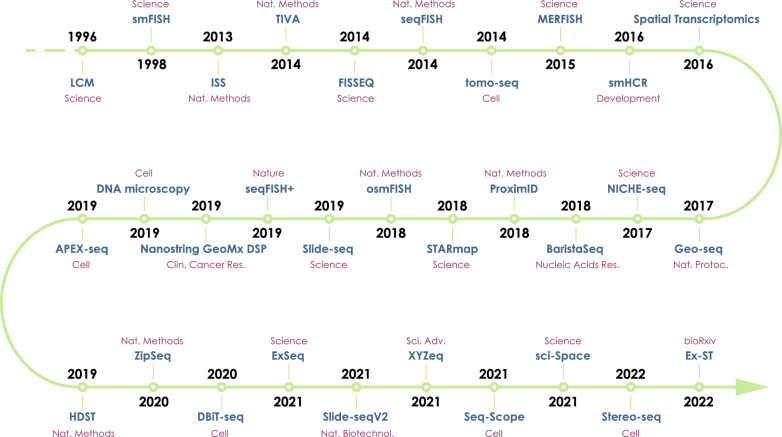
Development of spatial transcriptomics. The timeline indicates technologies (in bold blue), the years (in bold black) when the corresponding technologies were published and the journals (in dark red) where the corresponding technologies were published or employed (as in 10 × Genomics Visium). It should be noticed that scRNA-seq is presented in the figure only for reference, albeit a non-spatial technology. *LCM* Laser Capture Microdissection, *smFISH* Single-molecule RNA Fluorescence In Situ Hybridization, *ISS* In Situ Sequencing, *TIVA* Transcriptome In Vivo Analysis, *FISSEQ* Fluorescent In Situ RNA Sequencing, *seqFISH* Sequential Fluorescence In Situ Hybridization, *tomo-seq* RNA Tomography, *MERFISH* Multiplexed Error-robust Fluorescence In Situ Hybridization, *smHCR* Single-molecule Hybridization Chain Reaction, *Geo-seq* Geographical Position Sequencing, *BaristaSeq* Barcode In Situ Targeted Sequencing, *STARmap* Spatially-resolved Transcript Amplicon Readout Mapping, *osmFISH* Ouroboros Single-molecule RNA Fluorescence In Situ Hybridization, *DSP* Digital Spatial Profiling, *HDST* High-Definition Spatial Transcriptomics, *DBiT* Deterministic Barcoding in Tissue, *ExSeq* Expansion Sequencing, *Stereo-seq* Spatial Enhanced Resolution Omics-sequencing, *Ex-ST* Expansion Spatial Transcriptomics, *PNAS* Proceedings of the National Academy of Sciences of the United States of America, *Nat. Methods*Nature Methods, *Nat. Protoc.* Nature Protocols, *Nucleic Acids Res.* Nucleic Acids Research, *Clin. Cancer Res.* Clinical Cancer Research, *Nat. Neurosci.* Nature Neuroscience, *Nat. Biotechnol.* Nature Biotechnology, *Sci. Adv.* Science Advances

Since the initial spatial transcriptomics workflow was established in 2016 [5], this field has been proceeding apace with the unceasing evolution in resolution as well as throughput. Notably, spatially resolved transcriptomics was heralded as “Method of the Year 2020” by Nature Methods in 2021 [24]. Feasible methods for obtaining a fine-grained assessment of spatial transcriptome can be generally classified into four primary categories including microdissection, in situ hybridization, in situ sequencing, and spatial barcoding, each bearing its superiority and constraints. Overviews of these categories are summarized and a concise timeline depicting the remarkable course of spatial transcriptomics techniques is presented (Fig. 1) and detailed comparisons among existing methods are shown (Table 1). Some of the most commonly used spatial transcriptomics platforms are also listed in Table 2.

**Table 1:** Comparisons of Methods and Technologies for Spatial Transcriptomics

Technique	Year	Sample	Resolution	Genes detected	Strategy	Characteristic	Limitation	References
LCM	1996	Kidney glomeruli, Alzheimer's plaques, in situ breast carcinoma, etc	Cellular	N/A	Microdissection	Faster to perform No contamination to adjacent dissections	Low throughput	[25]
smFISH	1998	Normal rat kidney cells	Subcellular	2	In situ hybridization	Detects single transcripts High sensitivity	Low throughput	[29]
ISS	2013	Human breast cancer	Subcellular	31	In situ sequencing	Based on the padlock probe High accuracy	Needs pre-designed padlock probes	[38]
TIVA	2014	Mouse brain, human brain	Cellular	~ 9000	Spatial barcoding	Capture mRNA from live single cells in vivo	Low throughput	[72]
FISSEQ	2014	Human primary fibroblasts	Subcellular	8102	In situ sequencing	Transcriptome-wide RNA in situ sequencing	Low sequencing depth	[39]
seqFISH	2014	Yeast cells	Subcellular	12	In situ hybridization	Sequential barcoding Enables single-cell-resolution imaging of the transcriptome	Occurrence of errors that may be accumulated	[34]
tomo-seq	2014	Zebrafish embryo	N/A	~ 12,000	Microdissection	High sensitivity High spatial resolution. Construction of transcriptome-wide gene expression atlas in 3D	Several same biological samples needed	[27]
MERFISH	2015	Human fibroblast cells	Subcellular	140	In situ hybridization	Highly multiplexed Capable of detecting and correcting errors	Limited RNA measurement	[35]
smHCR	2016	Zebrafish embryos, mouse brain	Subcellular	5	In situ hybridization	High sensitivity Diffraction-limited resolution	Low throughput	[73]
Spatial Transcriptomics	2016	Adult mouse olfactory bulb	100 μm/55 μm (10 × Genomics Visium)	Entire transcriptome	Spatial barcoding	Provides spatial information	Contains several cells in each sequencing unit	[5]
Geo-seq	2017	Mouse early embryo, mouse brain, etc	10 cells	> 8000	Microdissection	Profiles transcriptomes from several cells while preserving spatial information	Low throughput	[28]
NICHE-seq	2017	Immune cells	Cellular	N/A	Microdissection	Elucidates spatial construction of cell types and corresponding molecular pathways	Limited to genetically engineered models	[74]
BaristaSeq	2018	Baby hamster kidney cells	Subcellular	N/A	In situ sequencing	High efficiency High accuracy	Needs pre-designed padlock probes	[75]
ProximID	2018	Mouse bone marrow	Cellular	N/A	Microdissection	Able to predict preferential associations between cells	Low throughput	[76]
STARmap	2018	Mouse primary visual cortex	Subcellular	160 ~ 1020	In situ sequencing	Able to measure the expression of a single cell in intact tissue High efficiency High accuracy	Low throughput	[42]
osmFISH	2018	Mouse brain	Subcellular	33	In situ hybridization	Automatically delineates tissue regions Able to process large tissue areas	Low throughput	[32]
Slide-seq	2019	Mouse brain	10 μm	Entire transcriptome	Spatial barcoding	High spatial resolution	Low capturing efficiency	[43]
seqFISH +	2019	Mouse brain, fibroblast cells	Subcellular	10,000	In situ hybridization	High accuracy Sub-diffraction-limit resolution	Low throughput	[77]
Nanostring GeoMx DSP	2019	Formalin-fixed, paraffin-embedded patient tissue	10 μm	N/A	Spatial barcoding	High-plex	May create bias in selecting regions	[78]
DNA microscopy	2019	MDA-MB-231 cells, BT-549 cells	Cellular	N/A	In situ sequencing	Able to image biological specimens without optical information Relies on thermodynamic entropy	Empty space causing sparse signals	[79]
APEX-seq	2019	HEK293T cells	Subcellular	N/A	Spatial barcoding	Performed in living cells Allows transcript isoforms with distinct localization to be distinguished	Limited application to human tissue	[80]
HDST	2019	Mouse olfactory bulb	2 μm	Entire transcriptome	Spatial barcoding	High resolution	Data sparsity	[45]
ZipSeq	2020	NIH/3T3 fibroblasts, live lymph node sections, mouse breast cancer	Cellular	Entire transcriptome	Spatial barcoding	Performed on live cells in intact tissues	Limited spatial resolution	[81]
DBiT-seq	2020	Mouse embryos	10 μm	22,969	Spatial barcoding	High spatial resolution Avoid lysis of tissues	Limited flow channels	[82]
ExSeq	2021	Mouse brain, human metastatic breast cancer	Subcellular	3039	In situ sequencing	High spatially precision Highly multiplexed imaging of RNAs in intact cells and tissues	Limits in detecting short transcripts	[40]
Slide-seqV2	2021	Mouse embryos, mouse brain	10 μm	1349	Spatial barcoding	High resolution Higher sensitivity than Slide-seq	May capture transcripts from multiple cells	[44]
XYZeq	2021	Human HEK293T cells, mouse NIH 3T3 cells	500 μm	Entire transcriptome	Spatial barcoding	Enables unbiased single-cell transcriptomic analysis	Requires specialized device	[83]
Seq-Scope	2021	Mouse liver and colon sections	~ 0.5–0.8 μm	Entire transcriptome	Spatial barcoding	High transcriptome capture efficiency Able to visualize the histological organization	Focused on only poly-A transcriptome	[46]
sci-Space	2021	Mouse embryos	200 μm	Entire transcriptome	Spatial barcoding	Retains single-cell resolution while capturing spatial information	Limited spatial resolution	[84]
Stereo-seq	2022	Mouse embryos, adult mouse brain and olfactory bulb	0.22 μm	Entire transcriptome	Spatial barcoding	High resolution High sensitivity Large visualizing field	Limited capturing efficiency	[85]
Ex-ST	2022	Mouse olfactory bulb and hippocampus	20 μm	Entire transcriptome	Spatial barcoding	Uses polyelectrolyte matrices to achieve higher resolution and detection efficiency	May capture transcripts from multiple cells	[86]

*smFISH* Single-molecule RNA Fluorescence In Situ Hybridization, *LCM* Laser Capture Microdissection, *ISS* In Situ Sequencing, *TIVA* Transcriptome In Vivo Analysis, *FISSEQ* Fluorescent In Situ RNA Sequencing, *seqFISH* Sequential Fluorescence In Situ Hybridization, *tomo-seq* RNA Tomography, *MERFISH* Multiplexed Error-robust Fluorescence In Situ Hybridization, *smHCR* Single-molecule Hybridization Chain Reaction, *Geo-seq* Geographical Position Sequencing, *BaristaSeq* Barcode In Situ Targeted Sequencing, *STARmap* Spatially-resolved Transcript Amplicon Readout Mapping, *osmFISH* Ouroboros Single-molecule RNA Fluorescence In Situ Hybridization. DSP, Digital Spatial Profiling, *HDST* High-Definition Spatial Transcriptomics, *DBiT* Deterministic Barcoding in Tissue. *ExSeq* Expansion Sequencing, *Stereo-seq* Spatial Enhanced Resolution Omics-sequencing, *Ex-ST* Expansion Spatial Transcriptomics

**Table 2:** Commonly used commercialized spatial transcriptomics technologies

Platform	Technique	Tissue Compatibility	Website
10 × Genomics Visium	ST	Fresh frozen, FFPE	https://www.10xgenomics.com/cn/products/spatial-gene-expression
Nanostring GeoMx DSP	DSP	Fresh frozen, FFPE	https://nanostring.com/products/geomx-digital-spatial-profiler/geomx-dsp-overview/
Vizgen MERSCOPE	MERFISH	Fresh frozen, FFPE	https://vizgen.com/products/

*FFPE* Formalin-fixed Paraffin-embedded, *DSP* Digital Spatial Profiling, *ST* Spatial Transcriptomics, *MERFISH* Multiplexed Error-robust Fluorescence In Situ Hybridization

### Technologies based on microdissection

Laser capture microdissection (LCM) [25] is a microdissection technique that employs a focused infrared laser pulse to isolate a specific tissue region of interest (ranging from 60 to 700 μm in diameter) from the original tissue section, enabling precise procurement of a specimen from the specified anatomical region while diminishing potential contamination. Moreover, these technologies are appropriate for partly-degraded tissue section analysis [26] and can interrogate the transcriptomes at a cellular resolution. One application of LCM technology is the genetic analysis of small premalignant lesions that have been isolated from histologically normal tissue or tumor edges, and this approach underlies several other technologies including tomo-seq [27], Geo-seq [28], etc.

Junker and colleagues [27] devised RNA tomography (tomo-seq), a technique that involves cryosectioning, reverse transcription, and amplification. Notably, this approach eliminates the need for carrier RNA and provides high sensitivity and spatial resolution. The robustness of the tomo-seq protocol was validated by the authors by applying it to zebrafish embryos, followed by a three-dimensional reconstruction of a genome-wide atlas at three developmental stages of the zebrafish embryo. The 3D profiling of tomo-seq was accomplished by cryosectioning three main body axes of the zebrafish and the data sets measured along these axes were reconstructed computationally by mapping gene expression information onto the image. Analysis of the 3D transcriptomic pattern of whole embryos and organs can be accomplished by tomo-seq but a main drawback of this method is that multiple samples are needed to generate sections of three axes so the application on human organs can be limited. Chen and colleagues [28] proposed another technology based on microdissection termed geographical position sequencing (Geo-seq) which integrates LCM and scRNA-seq technologies, enabling simultaneous investigation of cell heterogeneity and spatial variation. Geo-seq implements gene profiling at a ten-cell resolution, significantly facilitating the analysis of the spatiotemporally-regulated gene expression compared to individually utilizing the LCM method. In addition, Geo-seq can also promote the understanding of rare cells and the interaction between cells and surrounding niches. However, some impediments still remain, including the amplification merely of mRNA with a poly-A tail while preparing the library, which can be a hindrance for the subsequent Smart2-seq [28].

In summary, microdissection-based methods provide a competent approach to obtaining regions of interest from tissue samples with high sensitivity. These techniques enable focused research into the microanatomical structures and gene expression information of specific regions. However, Geo-seq, which integrates LCM and scRNA-seq (Smart2-seq), offers only a ten-cell resolution due to the limitations of microdissection-based techniques. During the laser-capturing and tissue segregation procedures of LCM, the quality of RNA molecules and the intactness of obtained cells may not be fully maintained. Additionally, microdissection is time-consuming and labor-intensive, limiting the throughput and the capacity to handle large tissue samples. Despite these shortcomings, microdissection-based technologies can still provide robust methods for gene expression profiling.

### Technologies based on in situ hybridization

In situ hybridization is a strategy that enables the visualization of RNA molecules within their original context via probes complementary to the objective transcripts rather than extracting them from tissue sections. An early iteration of in situ hybridization technique termed single-molecule fluorescent in situ hybridization (smFISH) [29] is competent in detecting several RNA transcripts simultaneously and has been advancing in gene measuring throughput and efficiency through multiplexed smFISH [30, 31]. This method exhibits high sensitivity and offers a subcellular resolution and is commonly utilized as a powerful tool for biological validation, such as corroborating the findings of bioinformatic analyses for newly identified genes. This technology requires fluorescent labeled RNA probes to hybridize with target molecules so the main drawback of smFISH is the limitation on the number of color channels due to the fluorescent overlapping of different channels, which means that smFISH can detect only a small number of genes concurrently. Another in situ hybridization technology called ouroboros smFISH (osmFISH) [32] is a non-barcoded and unamplified method based on cyclic smFISH, which can identify weakly-expressed genes [33] due to the circumvention of optical crowding. OsmFISH can be applied to large tissue samples, particularly for the examination of low-expression RNA transcripts. However, low throughput remains a technical limitation of this technique. Sequential FISH (seqFISH) is a barcoding protocol that leverages the high efficiency of FISH and the fact that distinguishing RNA transcripts does not require base-pair resolution [34]. In this approach, mRNAs are assigned temporal barcodes through multiple rounds of hybridization. During each round of hybridization, each transcript is targeted with several probes labeled with one color, and subsequently the probes are removed before the next round of hybridization where the same probes are labeled with fluorophores of a different color. Thus, seqFISH can generate a large number of transcripts while reducing spectral overlap that occurs in smFISH. However, seqFISH can be time-consuming and errors may accumulate over multiple rounds of hybridization, potentially leading to inaccurate information. Despite these limitations, seqFISH can be used to generate transcriptomic images of complex tissues, including brain samples [26].

To overcome the drawbacks of accumulating errors, Chen and colleagues [35] devised multiplexed error-robust FISH (MERFISH), a highly multiplexed smFISH protocol incorporating combinatorial labeling, successive rounds of sequential hybridization imaging, and error-robust encoding. MERFISH workflow is capable of measuring genes and combating accumulating detection errors by the error-robust encoding strategy designating each RNA transcript with a binary word. A 140-gene measurement was simultaneously performed with the encoding strategy that can detect and correct errors, whereas a 1001-gene measurement was performed with an alternative encoding strategy which can detect errors, albeit with no correction [35]. Notably, efforts have been made to evolve the MERFISH approach, enabling the simultaneous detection of RNA molecules to achieve up to 10,000 [36]. Moreover, MERFISH can be implemented to accomplish a high-throughput analysis of intercellular gene expression variation and elucidate the spatial distributions of multiple RNA transcripts concurrently. In contrast to seqFISH, the MERFISH protocol removes fluorophores but not the probes, making it more time-efficient than seqFISH [37]. The MERFISH approach has been commercialized as Vizgen MERSCOPE (Table 2) and can be applied to multiple tissue samples including fresh frozen and formalin-fixed paraffin-embedded (FFPE) tissue sections.

Overall, *in*-*situ*-hybridization-based techniques allow for the visualization of RNA molecules within their original tissue context by hybridizing probes with complementary targets. This enables the detection of target genes for biological validation of bioinformatic analysis results and the study of gene expression patterns. However, the nature of FISH methods imposes an intrinsic limitation on throughput. Additionally, specific probes must be synthesized before the hybridization process, necessitating the use of ready-made kits to overcome this challenge [33].

### Technologies based on in situ sequencing

In situ sequencing (ISS) method developed by Ke and colleagues [38] enables targeted analysis of RNA molecules in cells within a histomorphologically-retained context. This protocol entails single-strand DNA padlock probes with complementary sequences that bind to the cDNA generated by reverse transcription of mRNA molecules. Two targeted approaches, gap-targeted sequencing and barcode-targeted sequencing, were developed in the ISS procedure. In gap-targeted sequencing, the padlock probe has a gap between the probe ends which precisely binds to the targeted base pairs in the cDNA, and DNA polymerization and ligation subsequently fill the gap to form a circular DNA molecule. In barcode-targeted sequencing, the padlock probe contains a barcode sequence and only one breakpoint, so the formation of circular DNA undergoes only the ligation of the breakpoint. Rolling-circle amplification of the circularized DNA generates a rolling-circle product which then undergoes sequencing by ligation. The accuracy of the ISS protocol has been validated through its implementation in human breast cancer to manifest point mutations and decompose multiplexed gene expression profiling, using gap-targeted sequencing and barcode-targeted sequencing, respectively [38]. However, the ISS method requires prior knowledge of examined tissue to design padlock probes.

To examine transcripts without prior knowledge of tissue, Lee and colleagues [39] devised fluorescent in situ RNA sequencing (FISSEQ), a non-targeted approach measuring 8102 RNA species unbiasedly (transcriptome-wide). FISSEQ predominantly detects genes depicting cell type and function but low sequencing depth and incapability of ascertaining targeted RNA remain to be the drawbacks. Based on FISSEQ, another in situ sequencing strategy named expansion sequencing (ExSeq) was launched, enabling highly-multiplexed RNA visualization in cells and tissues of multiple-organ species with high spatial precision [40]. ExSeq encompasses targeted and untargeted versions, both of which can resolve biological problems ranging from nano-scale to system-scale. The targeted version addresses the issue of cellular crowding by attaching RNA molecules to an expandable hydrogel and expanding the hydrogel before ligating and sequencing, and the untargeted version optimizes the efficiency [41]. Untargeted ExSeq allows the detection of RNA molecules in the whole transcriptome including rare transcripts, whereas targeted ExSeq enables a smaller defined gene set to be detected and can be utilized to project cells onto tissue context and also visualize gene regulation. Wang and colleagues [42] developed spatially-resolved transcript amplicon readout mapping (STARmap) incorporating hydrogel-tissue chemistry and in situ sequencing, which can be employed to sequence RNA in 3D intact tissue with high efficiency and accuracy. Additionally notably, a modified STARmap scheme can be adopted for 3D analysis of thick tissue blocks, and sequencing with error-reduction by dynamic annealing ligation (SEDAL) was specifically devised for STARmap to eradicate misdecoding resulting from sequencing errors.

In contrast to traditional sequencing methods that separate cells from their spatial context, *in*-*situ*-sequencing-based methods enable spatial-level gene expression analysis and avoid the bias introduced by transcript extraction. However, these techniques still face challenges. For example, prior knowledge of the tissue may be required to design specific padlock probes, and read length may be limited. Additionally, in situ sequencing may not be feasible for unconventional or rare cell types and genes. Potential applications of these methods include studying gene expression regulation within tissues or cells and localizing gene variants.

### Technologies based on spatial barcoding

Ståhl and colleagues [5] proposed Spatial Transcriptomics (ST), which is practicable for quantitatively visualizing and determining the transcriptome whilst retaining spatial information. Tissue sections of adult mouse olfactory bulbs are placed on the glass slides immobilized with reverse transcription primers with poly-T to bind to the poly-A tail of mRNA derived from the tissue sections. The primers also embody spatial barcodes and unique molecular identifiers (UMIs) representing the coordinates of each array. During the tissue permeabilization process, mRNA molecules in tissue cells diffuse into 100-μm microwells on slides and hybridize with primers. Reverse transcription reagents are then added to the tissue to synthesize cDNA, using Cy3-labeled nucleotides for visualization of the generated cDNA. The tissue is subsequently removed by enzymes, leaving cDNA hybridized with nucleotides on the glass slides [5]. Although this technology provides spatial information, the resolution is limited to 100 μm, containing multiple cells. In 2019, 10 × Genomics further developed this method and commercialized it as “10 × Genomics Visium”, upgrading the resolution to 55 μm and refining the protocol to be compatible with both fresh frozen tissue sections and formalin-fixed paraffin-embedded (FFPE) tissue sections. This method has been widely used to study various tissue and disease. Maynard and colleagues [9] initially exploited the Visium platform to interpret gene expression information spatially in the human DLPFC on a transcriptomic scale.

Improvement of the resolution of spatial barcoding strategies has been continuously pursued. In 2019, Rodriques and colleagues [43] developed Slide-seq which provided an approach for spatially analyzing gene expression information at high resolutions (10 μm) analogous to the size of a single cell using beads deposited on the slide, with scalability to the large volume of tissue. Since these beads are randomly placed on the slide surface, their position information must be decoded through sequencing to match transcripts with their location, which may limit the capture efficiency. In 2021, Stickels and colleagues [44] described the improved version of Slide-seq, termed Slide-seqV2, which advanced approximately an order of magnitude in RNA capturing efficiency and sensitivity than the original Slide-seq. Not long after the publication of Slide-seq, a high-resolution spatial technology named high-definition spatial transcriptomics (HDST) utilizing barcoded bead arrays to capture RNA molecules from tissue sections in a histological context achieved a 2-μm resolution which is much higher than Spatial Transcriptomics [45]. It is also prominent that Seq-Scope technology yields a submicrometer resolution of 0.5 ~ 0.8 μm [46].

Slide-seq, HDST and Seq-Scope introduced above can provide much higher and even subcellular resolutions, generating more refined spatial distribution information. The approaches to improving the resolutions of Slide-seq and HDST are similar, involving bead arrays with 10-μm- and 2-μm-diameter beads, respectively [43, 45]. It should be noticed that Slide-seq and HDST involve beads similar to or smaller than the size of a single cell but they may cover multiple cells so the single-cell resolution may not be always achieved. Seq-Scope achieves subcellular resolution through the dense distribution of clustered barcodes. To be specific, many oligonucleotides containing high-definition map coordinate identifiers (HDMI) act as seed molecules, and an HDMI-array is generated by amplifying these seed molecules to form many clusters, each of which is derived from one seed molecule. This process can almost eliminate the areas with no detected RNA molecules [46]. However, pursuing such high resolution may introduce challenges such as data sparsity and difficulty inferring cell borders [47]. Noise is also a challenge due to limited coverage in each sequencing unit and the complex procedures required to maintain spatial positions during sequencing. The higher the resolution is, the more severe the noise is likely to be [48]. To improve the resolution while preserving comprehensive and necessary information, future breakthroughs may involve smaller but more sensitive detection units and the integration of spatial transcriptomics with high-throughput scRNA-seq data.

Overall, spatial-barcoding-based techniques allow for the simultaneous acquisition of gene expression and spatial location information. However, selecting the appropriate resolution requires careful consideration. Low resolution may obscure the intrinsic tissue structure and require further decomposition analysis to gain comprehensive insights, while high resolution may introduce those aforementioned challenges. Additionally, capture efficiency may be relatively low. Despite these limitations, spatial-barcoding-based techniques are widely used to study tissue architecture, tumor heterogeneity, the tumor microenvironment, etc.

## Gaining biological insights from spatial transcriptomics

**Fig. 2:**
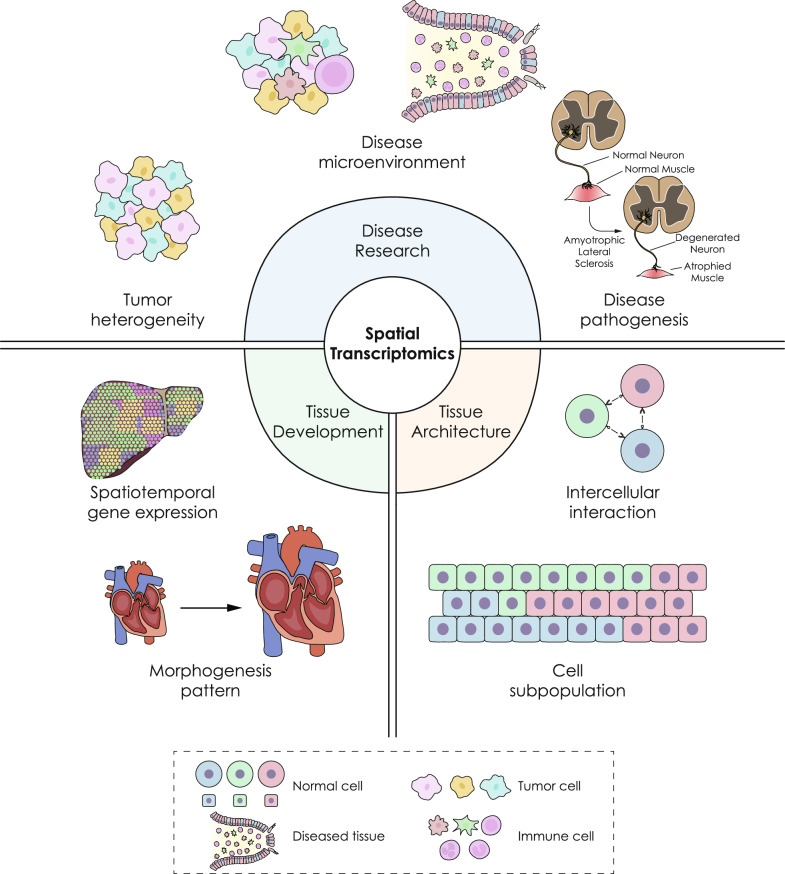
Application scenarios of spatial transcriptomics. “Tissue Architecture” refers to studies that elucidate the spatial distribution of cell subpopulations in a specific tissue and decode intercellular interaction. “Tissue Development” represents research into resolving morphogenesis patterns and spatiotemporal gene expression of the transcriptome during the development course of a certain tissue or organ. “Disease Research” demonstrates disease microenvironment and pathogenesis, and among the disease research, tumor biology is an important part including tumor microenvironment and heterogeneity

Spatial transcriptomics technologies are potent tools for studying the intricate structure, the dynamics of tissue and organ systems and inherent mechanisms within their original context. These technologies can provide valuable biological insights by revealing tissue architecture, developmental patterns and diseases, among which tumor biology may be one of the most extensive applications of spatial transcriptomics. Primary application scenarios of implementing spatial transcriptomics techniques are presented (Fig. 2) and several representative studies utilizing spatial transcriptomics are enumerated (Table 3).

**Table 3:** Representative applications utilizing spatial transcriptomics

Application	Tissue sample	Sequencing platform	Sample number	Journal	Author	Year	References
Tissue Architecture	Adult mouse brain	Illumina NextSeq	1	Sci Adv	Ortiz, C. et al.	2020	[6]
	Human postmortem DLPFC	10 × Genomics Visium	3	Nat Neurosci	Maynard, K.R. et al.	2021	[9]
	Wild type adult, female mouse livers	Illumina NextSeq500	8	Nat Commun	Hildebrandt, F. et al.	2021	[7]
	Human postmortem DRG	10 × Genomics Visium	8	Sci Transl Med	Tavares-Ferreira, D. et al.	2022	[8]
Tissue Development	Human embryonic heart	Illumina NextSeq	3	Cell	Asp, M. et al.	2019	[10]
	Adult mouse and adult human testis	Illumina NovaSeq S2	Mouse: N/A Human: 2	Cell Rep	Chen, H. et al.	2021	[13]
	Human embryonic intestine	Illumina NextSeq	5	Cell	Fawkner-Corbett, D. et al.	2021	[12]
	Human developmental liver	Illumina Hiseq3000	2	Front Cell Dev Biol	Hou, X. et al.	2021	[11]
Disease Research	Mouse spinal cord and postmortem spinal cord from ALS patient	N/A	Mouse: 67 Human: 7	Science	Maniatis, S. et al.	2019	[15]
	Mouse CD45^−^ lung cells after IAV infection and human lungs	Illumina NextSeq	Mouse: 4 Human: 3	Nature	Boyd, D.F. et al.	2020	[16]
	Human BPH specimen	10 × Genomics Visium & Nanostring GeoMx DSP	N/A	J Pathol	Joseph, D.B. et al.	2022	[14]
	Human heart	10 × Genomics Visium	31	Nature	Kuppe, C. et al.	2022	[17]
	Human lymph node metastases of stage III cutaneous malignant melanoma	Illumina NextSeq	4	Cancer Res	Thrane, K. et al.	2018	[21]
	Primary PDAC tumor	Illumina NextSeq	2	Nat Biotechnol	Moncada, R. et al.	2020	[18]
	Human cSCC	Illumina NextSeq	6	Cell	Ji, A.L. et al.	2020	[19]
	HER2-positive breast tumor	Illumina NextSeq500	8	Nat Commun	Andersson, A. et al.	2021	[20]
	Fresh hepatocellular carcinomas	10 × Genomics Visium	8	J Hepatol	Liu, Y. et al	2023	[49]
	OSCC and CRC	10 × Genomics Visium & Nanostring GeoMx DSP	OSCC: 1 CRC: 1	Nature	Galeano Niño, J.L. et al.	2022	[50]
	Early-stage lung cancer	Nanostring GeoMx DSP	12	J Immunother Cancer	Wong-Rolle, A. et al.	2022	[51]

*ST* Spatial Transcriptomics, *DRG* Dorsal Root Ganglia, *DLPFC* Dorsolateral Prefrontal Cortex, *ALS* Amyotrophic Lateral Sclerosis, *IAV* Influenza A Virus, *BPH* Benign Prostatic Hyperplasia, *DSP* Digital Spatial Profiling, *PDAC* Pancreatic Ductal Adenocarcinoma, *cSCC* Cutaneous Squamous Cell Carcinoma, *OSCC* Oral Squamous Cell Carcinoma, *CRC* Colorectal Cancer, *Sci Adv* Science Advances, *Nat Neurosci* Nature Neuroscience, *Nat Commun* Nature Communications, *Sci Transl Med* Science Translational Medicine, *Cell Rep* Cell Reports, *Front Cell Dev Biol* Frontiers in Cell and Developmental Biology, *J Pathol* Journal of Pathology, *Cancer Res* Cancer Research, *Nat Biotechnol* Nature Biotechnology, *J Hepatol* Journal of Hepatology, *J Immunother Cancer* Journal for Immunotherapy of Cancer

### Illustrating tissue architecture and developmental atlas

Decoding intercellular interaction and identifying cell subpopulations are of fundamental significance in delineating tissue architecture and defining structural components through the establishment of a transcriptome atlas of a specific tissue or organ, thus facilitating the perception of tissue dynamics. Hildebrandt and colleagues [7] managed to delineate the transcriptional landscape of sectioned mouse liver by employing spatial transcriptomics, corroborating the concept that liver lobular zonation characterized tissue heterogeneity by profiling of pericentral and periportal expression of representative marker genes. Ortiz and colleagues [6] accomplished a molecular atlas by applying spatial transcriptomics to a whole mouse brain to spatially manifest the brain tissue organization and composition. They also used a scRNA-seq dataset containing both neuronal and nonneuronal cells to map their spatial positions using a trained neural network model. This study demonstrates the potential of spatial transcriptomics to analyze complex samples such as brains, in addition to other tissues or organs. In addition, a study on the human dorsolateral prefrontal cortex (DLPFC) also resorts to spatial transcriptomics, which is notably the first research adopting the 10 × Genomics Visium platform, the commercialized version of spatial transcriptomics [9]. This study demonstrates the transcriptome-wide gene expression topography of human DLPFC across cortical laminae and subsequently a series of bioinformatics analyses are conducted to refine previous lamina-enriched genes and identify novel lamina-enriched genes. Moreover, the study delves into schizophrenia and autism spectrum disorder by incorporating previously-procured publicly-available neuropsychiatric disorder gene datasets to distinguish the particular lamina where genes associated with the diseases enrich, underlining the clinical significance of the study. Another study utilizing 10 × Genomics Visium probes into human nociceptors to present molecular features by applying the technology to human dorsal root ganglia [8]. Given that nociceptors are principal targets for acute and chronic pain treatment, the study might also provide insights into advancing medical treatment protocols and identifying novel drug targets.

Furthermore, spatial transcriptomics technologies are generally utilized in developmental biology to reveal spatiotemporal gene expression patterns and uncover tissue morphogenesis throughout the entire development course or multiple pivotal stages. Asp and colleagues [10] profiled a cell atlas of human cardiogenesis course where three developmental stages of the human embryonic heart were comprehensively delineated. They combined spatial transcriptomics with scRNA-seq to perform single-cell analysis and identify multiple cell types, and exploited in situ sequencing to position cells within their original clusters. The integration of spatial transcriptomics, scRNA-seq and in situ sequencing provides comprehensive insights into spatiotemporal patterns, marker genes, cellular interaction networks and developmental trajectories. Chen and colleagues [13] generated a spatial atlas for the transcriptome of mammalian spermatogenesis by adopting Slide-seq to mouse and human testis specimens and further characterized the microenvironment surrounding and mediating spermatogonial course by combining in situ sequencing.

### Disease research

Beyond the above insights about tissue architecture and development, spatial transcriptomics techniques have a robust capacity for clarifying disease microenvironments and pathogenesis. Boyd and colleagues [16] combined scRNA-seq with spatial transcriptomics to interrogate tissue inflammatory impairment in acute respiratory distress syndrome induced by severe respiratory influenza A virus infections. Their findings provided compelling evidence of the essential role played by lung fibroblasts in regulating immune reactions at the site of infections. This study demonstrates the utility of spatial transcriptomics in studying inflammatory diseases and the immune microenvironment and has stimulated research into immunopathy of other infectious diseases, including COVID-19, which continues to be a global health concern. Maniatis and colleagues [15] employed spatial transcriptomics on spinal cords from mice and amyotrophic lateral sclerosis patients to gain gene expression information to elucidate spatiotemporal dynamics mediating the degeneration of motor neurons. This research identifies the locations and distributions of specific genes associated with the disease and elucidates the underlying mechanisms regulating this neurodegenerative disorder.

A substantial part of disease research is the study of tumor biology which could be the most extensive application of spatial transcriptomics. Significant challenges in devising tumor treatment procedures are induced by tumor heterogeneity. Moncada and colleagues [18] utilized both scRNA-seq and spatial transcriptomics to investigate pancreatic ductal adenocarcinomas and distinguished cell populations and subsequently generated an unbiased map of the transcriptomes across the tumor, revealing its intrinsic architecture and heterogeneity. Another study that combined scRNA-seq and spatial transcriptomics to delineate the constitution and spatial architecture of cells within cutaneous squamous cell carcinoma revealed the cancer cell subpopulations and their communication [19]. The tumor microenvironment has become another hotspot of tumor-related research due to its complexity and diversity. Deciphering the tumor microenvironment is crucial for perceiving the intricate interactions between the tumor and microenvironment and may also aid in tumor immunotherapy. One study integrating spatial transcriptomics and scRNA-seq revealed the tumor microenvironment related to the immunotherapeutic efficacy of hepatocellular carcinoma, demonstrating a potential treatment target [49]. Another study analyzed the interactive relationship between the host and the microbiota in oral squamous cell carcinoma and colorectal cancer at a spatial level utilizing spatial transcriptomics and GeoMx digital spatial profiling [50]. It indicated that the tumor-associated microbiota, as an essential part of the tumor microenvironment, could impact tumor heterogeneity and induce the migration of cancer cells. Wong-Rolle and colleagues [51] conducted research related to intratumoral bacteria, where they discovered the enrichment of intratumoral bacteria in lung cancer and their association with several oncogenic pathways. The employment of spatial transcriptomics in tumor biology can reveal tumor heterogeneity and microenvironment to a large extent, thus providing ample instructions on addressing current obstructions confronting the treatment protocols.

## Data analysis of spatial transcriptomics

To comprehensively interrogate the tissue sections, bioinformatic analyses have to be performed to unravel the intertwined and multiplexed bioinformation and minimize the impact of current technological limitations and subsequently derive biological significance more accurately from raw spatial transcriptomics data. These bioinformatics analyses range from spatially-variable genes identification and clustering analysis to gene imputation, etc., which can be handily effectuated through a substantial number of computational strategies devised in recent years. Herein, circumstantial comparisons of algorithms and usages among the existing R or Python packages are presented (Table 4).

**Table 4:** Comparisons of computational strategies for spatial transcriptomics data analysis

Package name	Year	Journal	Developer	Algorithm	Programming language	Usage	Limitation	References
Seurat	2015	Nat Biotechnol	Satija, R. et al.	L1-constrained linear model	R	Clusters identification, data integration, gene imputation	Suitable for only certain platforms of ST	[22]
SpatialDE	2018	Nat Methods	Svensson, V. et al.	Gaussian process regression	Python	Spatially-variable genes identification	Heavy computational burden	[55]
trendsceek	2018	Nat Methods	Edsgärd, D. et al.	Marked point process	R	Spatially-variable genes identification	Heavy computational burden	[56]
GCNG	2020	Genome Biol	Yuan, Y. and Bar-Joseph, Z	Graph convolutional network	Python	Cellular interaction	Needs to be optimized when performed on individual datasets	[63]
SpaGE	2020	Nucleic Acids Res	Abdelaal, T. et al.	Domain adaptation model	Python	Data integration, gene imputation	Limited range of genes included in the model	[62]
SpaOTsc	2020	Nat Commun	Cang, Z. and Nie, Q	Structured optimal transport model	Python	Cellular interaction	Ignores time delay in cellular communication	[64]
SPARK	2020	Nat Methods	Sun, S. et al.	Generalized linear spatial model with penalized quasi-likelihood	R	Spatially-variable genes identification	Performs better for certain datasets but not all	[57]
SpatialCPie	2020	BMC Bioinformatics	Bergenstråhle, J. et al.	N/A	R	Clusters identification	Limited usage	[87]
stLearn	2020	bioRxiv	Pham, D. et al.	Transfer learning with a convolutional neural network, pseudo-space–time algorithm	Python	Clusters identification, cellular interaction, region annotation, spatial trajectories	Suitable for only certain platforms of ST	[69]
stereoscope	2020	Commun Biol	Andersson, A. et al.	Negative binomial distribution with maximum a posteriori estimation	Python	Data integration, spatial decomposition	Needs more deeply sequenced data	[88]
STUtility	2020	BMC Genomics	Bergenstråhle, J. et al.	Non-negative matrix factorization	R	Clusters identification, spatially-variable genes identification	Suitable for only certain platforms of ST	[52]
SPATA	2020	bioRxiv	Kueckelhaus, J. et al.	Shared-nearest neighbor clustering, pattern recognition, Bayesian model	R	Spatial trajectories, spatial CNV identification	Suitable for only certain platforms of ST	[67]
BayesSpace	2021	Nat Biotechnol	Zhao, E. et al.	Bayesian model with a Markov random field	R	Clusters identification	Suitable for only certain platforms of ST	[53]
DSTG	2021	Brief Bioinform	Song, Q. and Su, J	Semi-supervised graph-based convolutional network	Python	Data integration, spatial decomposition	Black-box problem of the Artificial Intelligence model	[89]
Giotto	2021	Genome Biol	Dries, R. et al.	A wide range of algorithms containing loess regression, HMRF, etc	R	Clusters identification, cellular interaction	Suitable for only certain platforms of ST	[90]
SOMDE	2021	Bioinformatics	Hao, M. et al.	Gaussian process	Python	Spatially-variable genes identification	Loss of some spatial details	[91]
MULTILAYER	2021	Cell Syst	Moehlin, J. et al.	Pattern recognition, community detection, agglomerative clustering	Python	Clusters identification, region annotation	May perform not as well on low-resolution data	[68]
SpaGCN	2021	Nat Methods	Hu, J. et al.	Graph convolutional network	Python	Clusters identification, spatially-variable genes identification, region annotation	Potential disagreement between actual tissue structure and detected spatial domains	[54]
SpatialDWLS	2021	Genome Biol	Dong, R. and Yuan, G.C	Weighted least squares	R	Data integration, spatial decomposition	Causes bias when removing some cell types	[61]
SPOTlight	2021	Nucleic Acids Res	Elosua-Bayes, M. et al.	Seeded non-negative matrix factorization regression	R	Data integration, spatial decomposition	Does not consider the information of capturing position	[59]
Tangram	2021	Nat Methods	Biancalani, T. et al.	Nonconvex optimization by a deep learning framework	Python	Data integration, spatial decomposition, gene imputation	Performs not as well on higher-density tissues	[23]
CARD	2022	Nat Biotechnol	Ma, Y. and Zhou, X	Conditional autoregressive model with a non-negative matrix factorization model	R	Data integration, spatial decomposition	Does not incorporate histology image	[58]
cell2location	2022	Nat Biotechnol	Kleshchevnikov, V. et al.	Bayesian model	Python	Data integration, spatial decomposition	Needs refinement for higher-resolution ST assays	[92]
CellTrek	2022	Nat Biotechnol	Wei, R. et al.	Coembedding and metric learning	R	Data integration, spatial decomposition	Sparse maps of cells in certain regions of tissue	[70]
RCTD	2022	Nat Biotechnol	Cable, D.M. et al.	Poisson distribution with maximum-likelihood estimation	R	Data integration, spatial decomposition	Disagreement of cell types between reference and spatial data	[60]
STAGATE	2022	Nat Commun	Dong, K. and Zhang, S	Graph attention auto-encoder	Python	Clusters identification, spatially-variable genes identification, gene imputation	Does not integrate histology images well	[93]
SpatialInferCNV	2022	Nature	Erickson, A. et al.	Hidden markov model	R	Spatial CNV identification	Does not capture SNV mutations or other copy-number-neutral events	[66]

*CARD* Conditional Autoregressive-based Deconvolution, *DSTG* Deconvoluting Spatial Transcriptomics Data Through Graph-based Convolutional Networks, *GCNG* Graph Convolutional Neural Networks for Genes, *RCTD* Robust Cell Type Decomposition, *SOM* Self-organizing Map, *DE* Differential Expression, *SPATA* SPAtial Transcriptomic Analysis, *SpaGCN* Spatial Graph Convolutional Network *SpaGE* Spatial Gene Enhancement, *SpaOTsc* Spatially Optimal Transporting the Single Cells, *SPARK* Spatial Pattern Recognition via Kernels, *DWLS* Dampened Weighted Least Squares, *STAGATE* Spatially Resolved Transcriptomics with an Adaptive Graph Attention Auto-encoder, *CNV* Copy Number Variation, *SNV* Single-nucleotide Variant, *ST* Spatial Transcriptomics, *HMRF* Hidden Markov Random Field, *Nat Biotechnol* Nature Biotechnology, *Nat Methods* Nature Methods, *Cell Syst* Cell Systems, *Genome Biol* Genome Biology, *Nucleic Acids Res* Nucleic Acids Research, *Nat Commun* Nature Communications, *Commun Biol* Communications Biology, *Brief Bioinform* Briefings in Bioinformatics

### Clusters identification

Distinguishing cell types and subpopulations is a fundamental task in the bioinformatic analysis of spatial transcriptomics data. This can be resolved with the help of clustering analysis where spatially-variable genes can be discovered and data dimensions can be reduced through approaches such as principal component analysis (PCA), t-distributed stochastic neighbour embedding (t-SNE) and uniform manifold approximation and projection (UMAP). These methods calculate similarity among barcode spots and define clusters within a tissue. A robust clustering procedure is provided by a widely-distributed R package Seurat [22], on which another R package capable of clustering analysis STUtility builds its framework [52]. Seurat is prevalent in scRNA-seq and spatial transcriptomics data analysis and is also competent in other bioinformatics analyses such as gene imputation. Zhao and colleagues [53] proposed BayesSpace based on a Bayesian model with a Markov random field, which outperformed previous clustering algorithms and improved spatial transcriptomics resolution to subspot levels. BayesSpace was validated by analyzing tissue samples, including brain and melanoma, overcoming challenges of low resolution and technical noise. SpaGCN is a python package based on a graph convolutional network that incorporates gene expression, spatial coordinates, and tissue histology visualization [54]. Clustering analysis is accomplished by aggregating gene expression from neighboring spots using a graph convolutional layer. SpaGCN has been tested on various species and utilized to analyze data generated from Spatial Transcriptomics and MERFISH. However, this strategy has the limitation of potential disagreement between actual tissue structure and detected spatial regions because the detection of spatial regions is primarily driven by gene expression information.

### Spatially-variable genes identification

Within a certain tissue, some genes exhibit conspicuous spatially-variable expression whereas some other genes such as housekeeping genes are expressed equally among the cells. The specific pattern in which the expressions of genes spatially vary can convey indispensable bioinformatic insights into identifying cell types and subpopulations and corresponding spatial information and underlying spatial functions. Some program packages perform outstandingly in identifying spatially-variable genes. Svensson and colleagues [55] described a strategy named SpatialDE, based on Gaussian process regression, which utilized two random effect models including a spatial variance model and a noise model to decompose variable expression of each gene into spatial and non-spatial components, respectively. Another package that identifies genes with statistical significance in spatial expression is termed trendsceek, building on marked point processes [56]. The trendsceek strategy can be performed on spatially resolved transcriptomics data sets and also scRNA-seq data projected onto a low dimension. Spatial pattern recognition via kernels (SPARK) technology, based on a generalized linear spatial model with a penalized quasi-likelihood algorithm, can overcome the high type I errors and low statistical power of previous strategies such as SpatialDE and trendsceek and is furthermore capable of analyzing large-scale spatial transcriptomics datasets [57]. However, SPARK may perform better for certain datasets and genes, causing intrinsic bias.

### Spatial decomposition and gene imputation

A common issue in spatial transcriptomics technology is that a single barcode-capturing spot may be overlaid by multiple cells. Thus, the detected expression is an aggregation of a heterogeneous set of cells within the spot, which may impact the efficiency and accuracy of identifying cell subpopulations and delineating tissue atlas. For example, 10 × Genomics Visium offers a resolution of 55 μm meaning the diameter of each capturing spot is 55 μm which is several-fold larger than a typical tissue cell. The spatial decomposition process through various deconvolution algorithms can address this discrepancy, which is to disentangle the mixture of mRNAs and subsequently predict the proportions of each cell type in one capturing spot. A spatial decomposition method devised by Ma and colleagues [58] is termed conditional autoregressive-based deconvolution (CARD) building on a non-negative matrix factorization model, which outperforms SPOTlight [59], RCTD [60], SpatialDWLS [61], etc. in deconvolution accuracy, corroborated by correlation analysis with scRNA-seq data. One potential improvement to this strategy is to incorporate tissue images, allowing for easier comparison between histological features and analysis results.

Gene imputation refers to the task of inferring lost gene expression information or “dropouts” caused by factors such as low protocol sensitivity, mitigating errors during gene measurement and facilitating deconvolution. Biancalani and colleagues [23] introduced a deep learning framework Tangram performing gene imputation. Gene imputation generated by Tangram yields an estimation of “dropouts” and prediction of spatial expression patterns more accurately conforming to MERFISH technology which is also competent in combating detection errors [35], thus promoting deconvolution of cells hampered by “dropouts”. The integrative and widespread R package Seurat can also impute gene expression utilizing co-expression patterns [22]. Abdelaal and colleagues [62] proposed Spatial Gene Enhancement (SpaGE) incorporating scRNA-seq and spatial data to predict gene expression which spatial transcriptomics techniques fail to detect, depending on a domain adaptation model. SpaGE is flexible and scalable when applied to large datasets and outperforms previous tools.

The aforementioned strategies, including spatial decomposition and gene imputation, have demonstrated considerable efficacy in enhancing the resolution of spatial transcriptomics data and compensating for lost gene expression information. Nevertheless, certain limitations persist. These approaches are based on computational models for predicting cell locations and gene information and therefore, their predictions may be subject to error, potentially resulting in imprecise and spurious results. Further investigation and refinement are necessary to more effectively leverage these technologies and derive more reliable biological insights.

### Cellular interaction

Cellular interaction operated within the microenvironment where cells are adjacent to each other can convey significant perceptions into tissue dynamics and the way the communication networks change when experiencing conditions such as disease. A Graph Convolutional Neural networks for Genes (GCNG) method was introduced to infer extracellular interactions from gene expression by depicting a cellular relationship graph transformed from spatial transcriptomics data and subsequently encoding gene expressions, and the graph is then convolved with expression information [63]. Cang and colleagues [64] launched spatially optimal transporting the single cells (SpaOTsc) to obtain intercellular communication, based on a structured optimal transport model. However, SpaOTsc does not account for time delays during intercellular communication. Owing to the three-dimensionality of tissue blocks, utilizing exclusively either scRNA-seq or spatial transcriptomics cannot output sufficient information to decipher cellular communication networks, therefore the integration of both datasets becomes a fundamental consideration when conducting bioinformatic analysis.

### Spatial copy number variations identification

Copy number variation (CNV) refers to the increase or decrease in the copy number due to gene segment rearrangements. Typically, CNVs involve segments longer than 1000 base pairs and are mainly manifested as submicroscopic deletions or duplications. CNVs are a common form of genetic variation in the human genome, with 5% ~ 10% of the genome affected by CNVs, which is much higher than other forms of genetic variation. Ascertaining the transition from benign to malignant tissue forms the foundation for improving early cancer diagnosis, as genomic instability in histologically benign tissue can signal an early event in cancer evolution. Furthermore, the spatial distribution and activity of CNVs can impact phenotype, making mapping their spatial distribution valuable for comprehending, diagnosing, and treating diseases. Previously, gene expression was utilized to infer CNVs in individual cells, successfully identifying regions of chromosomal gain and loss [65]. Erickson and colleagues [66] expanded this approach to a spatial modality with the development of SpatialInferCNV, an R package that identifies CNVs in each spatially barcoded region. Additionally, another package named SPATA also integrated a module for CNV detection [67].

### Region annotation and spatial trajectories

Gene expression within a tissue is influenced by the spatial position of cells in the tissue microenvironment. Spatial transcriptomic data can provide valuable insights into tissue regions, as they contain information on spatial position matrices, HE region staining of sections, and relative distances between individual cells, which can be used to delineate spatial regions. MULTILAYER is an algorithm that utilizes agglomerative clustering and community detection methods for graphical partitioning, enabling digital imaging of spatial transcriptomic analysis [68]. This allows for contextual gexel (namely, the locally defined transcriptomes) classification strategies, which can be used to develop self-supervised molecular diagnosis solutions.

Spatial trajectory analysis is an analytical method frequently employed in spatial transcriptomics to uncover dynamic cellular evolution and differentiation processes. This approach infers evolutionary trajectories and differentiation relationships between cells by analyzing their spatial positions and gene expression levels within tissue sections. The stLearn package can visualize spatial trajectories in tissue slices and infer biological processes from transcriptional state gradients across tissues [69]. Similarly, SPATA concentrates on temporal alterations in gene expression to deduce transcriptional patterns dynamically governed by the spatial organization [67].

### Data integration

Both spatial transcriptomics and scRNA-seq are effective methods for obtaining biological insights into tissues and diseases. However, each method has its limitations. By integrating spatial transcriptomics and scRNA-seq data, these methods can complement each other to provide comprehensive biological information. For instance, RCTD generates spatial decomposition by assigning cell types to spatial transcriptomics spots [60], whereas Tangram performs gene imputation by aligning scRNA-seq data with spatial transcriptomics data to learn spatial transcriptome-scale paradigm [23]. Additionally, CellTrek is a computational strategy that integrates scRNA-seq and spatial transcriptomics data sets to perform spatial decomposition by reconstructing a cellular map on tissue sections [70]. This strategy is distinct from other spatial decomposition methods in that CellTrek directly maps single cells to corresponding spatial positions in the spatial context. Other than these R or Python packages, many studies have incorporated spatial transcriptomics and scRNA-seq. Liu and colleagues [49] discovered a tumor immune barrier structure and a series of cancer-associated fibroblasts related to the efficacy of immune treatments through an integrative analysis of spatial transcriptomics and scRNA-seq. The scope of ‘data integration’ encompasses not only the alignment of these two methods but also the incorporation of spatial transcriptomics with other omics data. However, few individual computational tools are designed specifically for combining spatial transcriptomics and other omics. Therefore, linking multiple packages for analysis is necessary. For instance, a remarkable study integrated spatial transcriptomics, scRNA-seq, proteomics and whole-exome sequencing to resolve pancreatic cancer microenvironment, utilizing various packages including Seurat, RCTD, CellPhoneDB (for detecting ligand-receptor interactions), Monocle3 (for inferring cell transitions), inferCNV (for detecting CNVs in scRNA-seq data), germlinewrapper and somaticwrapper (for calling germline variants and somatic variants, respectively), among others [71]. Thus, we can see the significant potential in the integrative analysis of spatial transcriptomics, scRNA-seq and other omics.

### A brief pipeline of spatial transcriptomics data analysis

Methods for analyzing spatial transcriptomics data are generally similar and can be divided into data preprocessing and downstream analysis. Data preprocessing typically involves quality control and normalization to improve data quality for downstream analysis and obtain more reliable biological information. For spatial-barcoding-based methods, quality control aims to remove low-quality spots and genes from spatial transcriptomics data. Quality control parameters can be adjusted based on tissue type, research requirements, and other factors. These parameters may include removing spots with fewer than a certain number of transcripts, removing genes expressed in fewer than a certain number of spots, and removing spots with a high proportion of mitochondrial genes. Normalization accounts for the difference in sequencing depth among different spots. Since differences among spots in spatial transcriptomics data can be relatively large, effective normalization is essential.

After preprocessing, downstream analysis can be performed. The data should first undergo dimensionality reduction and clustering analysis to distinguish spots with different features. Biological information can then be interpreted through these clusters in subsequent analysis. Algorithms such as PCA, t-SNE, and UMAP can be used for this purpose and are available in many data analysis packages. Next, gene expression patterns in the data can be analyzed, including differential expression analysis and spatially variable gene analysis, which can be performed using packages such as Seurat and SpatialDE, respectively. Additionally, cell information from tissue slices can be annotated onto spatial transcriptomics data. Since the sequencing unit (e.g., spots in 10 × Genomics Visium and beads in Slide-seq) of some spatial transcriptomics technologies may contain more than one cell, spatial decomposition can infer the proportion of various cells in each sequencing unit based on the data to obtain cell locations in the spatial context. This step can be achieved using packages with deconvolution algorithms such as RCTD and cell2location. Gene imputation can also predict the positions of low-expressed or missing genes in space due to possible dropout using packages like Tangram. Furthermore, personalized analysis can be conducted based on research objectives. For instance, packages such as Giotto can be used to analyze the communication between cells or spatial regions, including receptor-ligand interactions. SpatialInferCNV can perform copy number variation analysis at the spatial level, while stLearn and SPATA can be used for spatial trajectory analysis and MULTILAYER for spatial region identification. These analytical methods and packages provide excellent visualization during data analysis, facilitating step-by-step comprehension of current analytical outcomes to guide subsequent analysis. Moreover, it is essential to integrate spatial transcriptomics data with scRNA-seq data and other omics data to obtain a more comprehensive understanding of biological information.

## Conclusion and future perspectives

Explosive advances in spatial transcriptomics technologies have been made in recent years to expand our understanding of miscellaneous tissues and organs. However, current spatial transcriptomics methods are confronted with some challenges of low resolution, sensitivity, throughput, etc., hindering our precise perception of normal and abnormal tissues, which calls for further innovations in technologies to overcome these deficiencies. Given that each technology bears its biological strengths, we envision the integration across these technologies which complement each other in the drawbacks before a novel and robust technology is launched. With future technology revolutions, intercellular signaling could be resolved at higher and even single-cell resolution. In addition, larger-scale tissue specimens may be investigated to allow for depicting organ-level tissue topography, enabling a more holistic and consecutive interpretation of tissue structures, which latently poses challenges for accelerating bioinformatic analysis with higher efficiency and accuracy and more powerful information processing capacity. Beyond the prospective advancement in refining and optimizing current protocols of spatial transcriptomics, we also envisage the integration with multi-omics including epigenomics, proteomics, and metabolomics to shed light on the intrinsic convoluted mechanisms of cellular interactions and disease and better probe into tumor progression and growth course. In addition to advances in spatial transcriptomics technologies, innovations in data analysis strategies are also anticipated. As deep learning technology continues to progress, its application in spatial transcriptomics data analysis is expected to become more widespread. In the future, more deep-learning-based methods may be developed to process and analyze spatial transcriptomics data to improve data resolution and interpretation reliability. Furthermore, as data scale and complexity increase, visualization and interactive analysis will become important tools for spatial transcriptomics data analysis. Future spatial transcriptomics data analysis methods will need to integrate visualization and interactive analysis technologies to better understand and interpret data.

Since some spatial transcriptomics techniques, especially some widespread spatial-barcoding-based techniques, are not capable of offering single-cell resolution at the spatial level and scRNA-seq cannot reflect the spatial distribution of each cell, we envision a more organic and efficient alignment of single-cell datasets and corresponding spatial information. The alignment can be achieved by mapping single cells to spatial data, where each cell is matched with a spatial location in an ideal condition. Nevertheless, current methods for integration cannot generate precise matching due to technological limitations, which calls for further breakthroughs in the effectiveness and efficiency of data integration algorithms. By integrating both datasets, we can decipher potential intercellular communication pathways, including ligand-receptor interactions and juxtacrine and paracrine signaling. This may provide insights into previously unclear physiological and disease mechanisms and help discern more refined classifications of certain diseases, facilitating precise and individualized medical treatment. Additionally, publicly-available datasets can be interrogated retrospectively with the integration of spatial transcriptomics and scRNA-seq data to obtain novel biological cues which may be concealed in the raw data before.

Moreover, we anticipate the translational medicine research into the clinical significance of spatial transcriptomics, particularly with the compatibility of the 10 × Genomics Visium platform with FFPE tissue blocks allowing retrospective analysis into previously opaque tissue specimens to glean more sufficient information on clinical diagnostics and prognostics as well as therapeutic methods and targets. For example, research into human DLPFC distinguished the layer-enriched genes that may be associated with schizophrenia and autism spectrum disorder, implicating the potential of neuropsychiatric disorders progression in those bearing the risk gene expression [9]. In tumor biology, spatial transcriptomics incorporated with other omics can identify cancer gene signatures and subsequently reveal novel targets for cancer treatment and assist us to abate or suppress the degree of tumor cell proliferation, infiltration, and invasion. Nevertheless, it is noteworthy that before translating omics data into clinical relevance, the robustness of the technologies and the quality of specimens and specimens processing must be considered.

## Data Availability

Not applicable.

## Abbreviations

smFISH: Single-molecule RNA fluorescence in situ hybridization
LCM: Laser capture microdissection
scRNA-seq: Single-cell RNA sequencing
ISS: In situ sequencing
TIVA: Transcriptome in vivo analysis
FISSEQ: Fluorescent in situ RNA sequencing
seqFISH: Sequential fluorescence in situ hybridization
tomo-seq: RNA tomography
MERFISH: Multiplexed error-robust fluorescence in situ hybridization
smHCR: Single-molecule hybridization chain reaction
Geo-seq: Geographical position sequencing
BaristaSeq: Barcode in situ targeted sequencing
STARmap: Spatially-resolved transcript amplicon readout mapping
osmFISH: Ouroboros single-molecule RNA fluorescence in situ hybridization
DSP: Digital spatial profiling
HDST: High-definition spatial transcriptomics
DBiT: Deterministic barcoding in tissue
ExSeq: Expansion sequencing
Stereo-seq: Spatial enhanced resolution omics-sequencing
Ex-ST: Expansion spatial transcriptomics
UMI: Unique molecular identifiers
FFPE: Formalin-fixed paraffin-embedded
DLPFC: Dorsolateral prefrontal cortex
COVID: Corona virus disease
ST: Spatial transcriptomics
DRG: Dorsal root ganglia
ALS: Amyotrophic lateral sclerosis
IAV: Influenza A virus
BPH: Benign prostatic hyperplasia
PDAC: Pancreatic ductal adenocarcinoma
cSCC: Cutaneous squamous cell carcinoma
CARD: Conditional autoregressive-based deconvolution
DSTG: Deconvoluting spatial transcriptomics data through graph-based convolutional networks
GCNG: Graph convolutional neural networks for genes
RCTD: Robust cell type decomposition
SOM: Self-organizing map
DE: Differential expression
SPATA: Spatial transcriptomic analysis
SpaGCN: Spatial graph convolutional network
SpaGE: Spatial gene enhancement
SpaOTsc: Spatially optimal transporting the single cells
SPARK: Spatial pattern recognition via kernels
DWLS: Dampened weighted least squares
STAGATE: Spatially resolved transcriptomics with an adaptive graph attention auto-encoder
CNV: Copy number variation
HMRF: Hidden markov random field
PCA: Principal component analysis
t-SNE: T-distributed stochastic neighbour embedding
UMAP: Uniform manifold approximation and projection
